# Neonatal outcomes of deliveries in occiput posterior position when delayed pushing is practiced: a cohort study

**DOI:** 10.1186/s12884-017-1556-5

**Published:** 2017-11-14

**Authors:** Kristina Dahlqvist, Maria Jonsson

**Affiliations:** 1 Department of Women’s and Children’s Health, Uppsala University, Uppsala, Sweden; 2 Department of Obstetrics and Gynaecology, Örnsköldsvik Hospital, SE-891 89 Örnsköldsvik, Sweden

**Keywords:** Acidaemia, Apgar score, Caesarean delivery, Delayed pushing, Metabolic acidaemia, Labour, Neonatal morbidity, Occiput anterior position, Occipito-posterior position, Occiput posterior position

## Abstract

**Background:**

To examine the impact of occiput posterior position, compared to occiput anterior position, on neonatal outcomes in a setting where delayed pushing is practiced. The specific aim was to estimate the risk of acidaemia.

**Methods:**

Cohort study from a university hospital in Sweden between 2004 and 2012. Information was collected from a local database of 35,546 births. Umbilical artery sampling was routine. Outcomes were: umbilical artery pH < 7.00 and <7.10 and short-term neonatal morbidity. The association between occiput posterior position and neonatal outcomes was examined using logistic regression analysis, presented as adjusted odds ratio (AOR) with 95% confidence interval (CI).

**Results:**

Of 27,648 attempted vaginal births, 1292 (4.7%) had occiput posterior position. Compared with occiput anterior, there was no difference in pH < 7.00 (0.4% vs. 0.5%) but a higher rate of pH < 7.10 in occiput posterior births (3.8 vs. 5.5%). Logistic regression analysis showed no increased risk of pH < 7.10 (AOR 1.28 95% CI 0.93–1.74) when occiput posterior was compared with occiput anterior births but, an increased risk of Apgar score < 7 at 5 min (AOR 1.84, 95% CI 1.11–3.05); neonatal care admission (AOR 1.68, 95% CI 1.17–2.42) and composite morbidity (AOR 1.66, 95% CI 1.19–2.31).

**Conclusions:**

With delayed pushing, birth in occiput posterior compared with anterior position is not associated with acidaemia. The higher risk of neonatal morbidity is of concern and any long-term consequences need to be investigated in future studies.

## Background

Persistent occiput posterior position occurs in approximately 5% of births and is the most common malposition in labour [1–5]. Due to incomplete flexion of the fetal head with occiput posterior positon, resulting in larger presenting diameter, delivery can be expected to be more difficult. Previous studies have reported an association with longer labour duration [1–5], oxytocin augmentation [1, 3, 4], epidural analgesia [1, 3–5], operative vaginal delivery [1–4], severe perineal laceration [3, 4], and caesarean delivery [1, 3–5]. Neonatal outcomes may, consequently, also be influenced and an increased risk of Apgar score < 7 at 5 min, birth trauma, admission to neonatal care, and cord acidaemia has been reported [1, 6].

In observational studies, occiput posterior delivery has been found to be a risk factor for acidaemia at birth [1, 6]. The risk of having an umbilical artery pH < 7.00 increased three fold in occiput posterior, compared with occiput anterior delivery, at a unit with a policy of immediate pushing once full cervical dilation is reached [1]. There is an increase in the incidence of serious neonatal morbidity with an umbilical artery pH < 7.00 [7, 8], giving this finding important clinical implication.

Management of the second stage of labour when occiput posterior position occurs is critical; however, clinical guidance in the literature is sparse. In general, management of the second stage of labour is subject to divergent approaches, and controversies exist regarding when the woman should begin to push. In delayed pushing, passive descent is awaited and pushing initiated after a time interval, or when there is an urge to push. In immediate pushing, women are encouraged to push when complete cervical dilation occurs. Compared with immediate pushing, delayed pushing is associated with higher rates of spontaneous vaginal deliveries [9, 10], a reduction of operative vaginal deliveries [9, 10], a longer second stage, but decreased duration of pushing [10, 11] and no differences in neonatal outcomes [10–12].

The aim of this study was to investigate the influence of occiput posterior position on neonatal outcomes when delayed pushing was practiced. The primary aim was to estimate the risk of acidaemia and short-term neonatal outcomes, comparing occiput posterior position with occiput anterior position.

## Methods

The study was designed as a retrospective cohort study of all attempted vaginal deliveries between the years 2004 and 2012 at the University Hospital in Uppsala, Sweden. The hospital is a referal centre with 4000 deliveries annually. Data were obtained from an established perinatal database. Exclusion criteria were: gestational length less than 37 weeks; multiple gestation; a history of previous caesarean delivery; caesarean delivery before labour onset in the current pregnancy; non-cephalic presentation; face or brow presentation; and stillbirths. A validation of the database concerning occiput posterior position showed that in 100 selected births (every tenth) recorded as occiput posterior, 100% were occiput posterior according to individual records.

The regional Ethical Committee in Uppsala approved the study (Dnr 2012/410).

The midwife responsible for the delivery recorded maternal demographics, labour characteristics including fetal position at delivery, and neonatal information in the database. A normal pregnancy was defined as no observed complication to the pregnancy, such as diabetes, hypertension, preeclampsia, premature rupture of membranes (> 24 h), fetal growth restriction, oligohydramniosis, post term pregnancy (≥ 42 gestational weeks), intrahepatic cholestasis, red blood cell allo-immunization, or other complications.

Either certified nurse-midwives managed deliveries autonomously or, in case of abnormal labour, in association with the attending obstetrician, or by resident physicians, under the supervision of a senior colleague. The approach to the second stage of labour is passive fetal descent i.e. waiting for the urge to push. In case of epidural analgesia and absent urge to push, the woman is instructed to push when the fetal head has descended to the pelvic floor. Umbilical cord acid base analysis (artery and vein) is routinely attempted in all deliveries. Vacuum-assisted vaginal delivery is the mode of operative vaginal delivery and forceps are not used. In case of caesarean delivery, an attending midwife registered the fetal presentation. There was no information on manual rotation manoeuvres of occiput posterior positions.

A fetus delivered in occiput posterior position (right, direct and left) defined persistent occiput posterior position. The association between occiput posterior position at birth and neonatal outcomes was investigated using the occiput anterior position as reference. The primary outcome variable was acidaemia at birth, defined as umbilical artery pH < 7.00. Secondary outcome variables were umbilical artery pH < 7.10, < 7.05, Apgar score < 7 at 5 min, admission to neonatal intensive care unit and composite morbidity. The composite variable included one or more of: umbilical artery pH < 7.00; metabolic acidaemia (pH < 7.00 and base deficit > 12 mmol/L); an Apgar score < 7 at 5 min; or referral to neonatal care.

Maternal age was categorized into less than 35, or 35 years and older. Birth weight was categorized into < 3295 g, 3295–3925 g, and > 3925 g, based on the 25th and 75th centile of the study population. To investigate the influence of position on mode of delivery and neonatal outcomes, the deliveries were stratified into spontaneous, operative vaginal, and caesarean delivery. The delivery years were divided in two time intervals: 2004–2007 and 2008–2012, to eliminate possible confounding effects of changes in obstetric care during the study period.

We calculated the risk in each group (occiput posterior and anterior) and the risk difference between groups with 95% confidence interval (CI). The 95% CI for the risk was calculated using the Asymptotic Score method and the 95% CI for the risk difference was calculated using the Newcombe method. For adjusted analysis, the Generalized Estimating Equations logistic regression model was used to assess the association between occiput posterior position and neonatal outcomes. Odds ratios were adjusted for maternal age, nulliparity, pregnancy complication, induction of labour, gestational age at birth, use of epidural analgesia, birth weight and year of delivery. Calculations were only made on deliveries with complete registration of data and if the number of events in the delivery mode subgroup were at least 60. A sensitivity analysis with exclusion of birth weight and gestational age was done and these variables did not affect the results. An exchangeable correlation structure was used to control for the dependence between births by the same woman. To compare group distributions, the Chi 2-test was applied for categorical variables, and the Student *t* test or the Mann-Whitney-U test for continuous variables.

To examine the primary outcome, umbilical artery pH less than 7.00, with a baseline rate of 0.5% and to find at least 1% difference between occiput posterior (5%) and occiput anterior positon, our sample size of > 27,000 births was estimated to have 75% power and with 1.3% difference we would have 87% power (alpha 0.05). Rates of umbilical artery pH less than 7,00 that were found in the study by Cheng et al. were used for power calculation; 0.5% in occiput anterior and 1,8% in occiput posterior position [1]. All analyses were performed using the Statistical Analysis Software, version 9.2 (SAS Institute, Cary, NC).

## Results

**Fig. 1:**
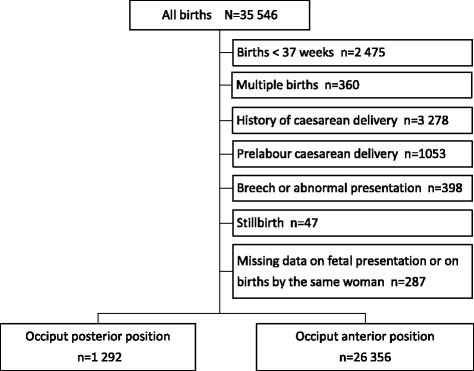
Flow diagram of the study population

During the study period there were 35,500 births. After exclusion of 7852 (22%) births, 27,648 term, singleton, cephalic live births remained and formed the study population. Figure 1 displays a flow diagram of the studypopulation. The incidence of persistent occiput posterior position was 4.7% (*n* 1292). Umbilical cord blood sampling was successful in 74%, with no difference in sampling rate between vaginal deliveries in occiput posterior (76%), and anterior (73%) positions (*p* 0.06) whereas there were more samples performed in deliveries in the occiput posterior position if all deliveries were considered (79% vs. 74%, *p* < 0.001).

Presented in Table 1 are maternal demographics and labour characteristics. A higher rate of occiput posterior position was found both among nulliparous women and among those with a gestational age of 41 weeks or more. Labours with occiput posterior position required oxytocin and epidural analgesia more often. The pushing phase was longer with occiput posterior, compared to anterior (mean of 51 vs. 39 min, (*p* 0.003), and lasted more than 45 min in 35% of occiput posterior, compared to 21% with anterior position (*p* < 0.001). The higher rate of missing values for duration of pushing in occiput posterior position deliveries can be explained by the higher rate of caesarean deliveries in that group. Spontaneous vaginal delivery occurred in 86% of anterior, compared to 49% of occiput posterior (*p* < 0.001), and caesarean was performed in 5.6% compared to 35.6% (*p* < 0.001).

**Table 1:** Maternal demographics and labour characteristics

Variable	All *N* = 27,648	Occiput posterior *N* = 1292	Occiput anterior *N* = 26,356	*P* value
Maternal age (years)				
Mean (SD)	30.7 (5.0)	30.6 (5.0)	30.7 (5.0)	0.53
≥ 35	5404 (19.5)	251 (19.4)	5153 (19.6)	0.96
Nulliparity	12,806 (46.3)	750 (58.1)	12,056 (45.7)	< 0.001
Missing	6 (0.02)	2 (0.15)	4 (0.02)	
Gestational length				
Mean (SD), days	280 ± 8	281 ± 9	280 ± 8	0.005
< 41 weeks	19,836 (71.7)	885 (68.5)	18,951 (71.9)	0.008
Normal pregnancy	20,950 (85.2)	925 (80.4)	20,025 (85.5)	< 0.001
Missing	3064 (11)	141 (11)	2923 (11)	
Induction of labour	4018 (14.6)	247 (19.2)	3771 (14.4)	< 0.001
Missing	198 (0.7)	7 (0.5)	191 (0.7)	
Oxytocin				
In the first stage of labour	7436 (26.9)	599 (46.4)	6837 (25.9)	< 0.001
In the second stage of labour	7880 (28.5)	483 (37.4)	7397 (28.1)	< 0.001
First and second stage	4255 (15.4)	258 (20.0)	3997 (15.2)	< 0.001
Epidural analgesia	9276 (33.6)	620 (48.0)	8656 (32.8)	< 0.001
Duration of pushing (min)				
Mean (SD)	39.0 (113.8)	51.4 (123.1)	38.6 (113.5)	0.003
> 45 min	4947 (21.7)	255 (35.1)	4692 (21.2)	< 0.001
Missing	4811 (17)	565 (44)	4246 (16)	
Mode of delivery				
Spontaneous vaginal	23,323 (84.4)	635 (49.1)	22,688 (86.1)	< 0.001
Operative vaginal	2400 (8.7)	197 (15.2)	2203 (8.4)	< 0.001
Caesarean section	1924 (7.0)	460 (35.6)	1464 (5.6)	< 0.001
Missing	1	0	1	
Birth weight (g)				
Mean (SD)	3620 (487.2)	3645 (508.5)	3619 (486.1)	0.07
< 3295	6770 (25.3)	316 (25.0)	6454 (25.3)	0.80
3295–3925	13,242 (49.4)	603 (47.7)	12,639 (49.5)	0.24
> 3925	6794 (25.3)	344 (27.2)	6450 (25.3)	0.11
Missing	842 (3)	29 (2)	813 (3)	

Data are presented as *n* (%) or mean ± standard deviation (SD)

Neonatal outcomes are displayed in Table 2. Occiput posterior, compared to anterior position had a higher incidence of umbilical artery pH < 7.10 and no difference in incidence of pH < 7.05 or < 7.00. Mean pH (± standard deviation) differed: 7.23 ± 0.07 vs. 7.25 ± 0.07 in occiput posterior and occiput anterior position, respectively (*p* < 0.001), data not shown in table). Compared with anterior, neonates in occiput posterior position had a higher incidence of Apgar score < 7 at 5 min (1.5% vs. 0.7% with a risk difference of 0.83, 95% CI 0.25–1.71). The incidence of composite morbidity was higher in occiput posterior (3.6%) compared to anterior (2.0%), *p* 0.03. The major contributor to the composite morbidity was admission to neonatal care. In the analysis stratified by mode of delivery (data not shown in table) there was no case with Apgar score < 7 at 5 min in spontaneous vaginal delivery in occiput posterior, compared with 70 (0.3%) cases in anterior, with a risk difference of −0.31 (95% CI -0.39-0.44). Operative vaginal delivery in occiput posterior position resulted in 7 (3.6%) cases with Apgar score < 7 at 5 min, compared to 53 (2.4%) cases with the anterior position; the risk difference was 1.14 (95% CI -0.99 to 5.11).

**Table 2:** Neonatal outcomes in occiput posterior and anterior births and the risk difference between the groups

Outcome	ALL *N* = 27,648	Occiput posterior *N* = 1292		Occiput anterior *N* = 26,356		Risk difference
	*n* (%)	*n* (%)	95% CI	*n* (%)	95% CI	95% CI
Umbilical artery pH						
< 7.00	88 (0.4)	5 (0.5)	0.21–1.14	83 (0.4)	0.34–0.53	0.06 (−0.26–0.78)
< 7.05	278 (1.4)	16 (1.6)	0.96–2.53	262 (1.3)	1.19–1.52	0.22 (−0.44–1.25)
< 7.10	789 (3.9)	56 (5.5)	4.24–7.04	733 (3.8)	3.51–4.05	1.71 (0.40–3.35)
Metabolic acidaemia^a^	35 (0.2)	2 (0.2)	0.05–0.72	33 (0.2)	0.12–0.24	0.03 (−0.15–0.62)
Apgar < 7 at 5 min	208 (0.8)	20 (1.5)	1.00–2.38	188 (0.7)	0.62–0.83	0.83 (0.25–1.71)
Neonatal care	458 (1.7)	38 (2.9)	2.15–4.01	420 (1.6)	1.45–1.75	1.35 (0.51–2.47)
Composite morbidity^b^	567 (2.1)	47 (3.6)	2.75–4.80	520 (2.0)	1.81–2.15	1.66 (0.72–2.89)

Data are presented as n (%) with 95% confidence interval (CI). Calculations were only made on deliveries with complete registration of data

^a^Metabolic acidaemia: umbilical artery pH < 7.00 and base deficit ≥ 12 mmol/L

^b^Composite morbidity: any of Apgar score < 7 at 5 min; pH < 7.00; metabolic acidaemia or admission to neonatal care

Table 3 illustrates the result of the logistic regression analysis of associations between occiput posterior position and neonatal outcomes. Compared with occiput anterior, occiput posterior position had no association to pH < 7.10 or a risk of metabolic acidaemia at birth. Occiput posterior position increased the risk of Apgar score < 7 at 5 min with 84%, the need for neonatal care with 68% and of composite morbidity with 66%, compared to occiput anterior.

**Table 3:** Logistic regression analysis to assess the association between occiput posterior position and neonatal outcomes, all births

Outcome	All births			
	*n*	Rate	OR (95% CI)	AOR^a^ (95% CI)
Umbilical artery pH < 7.10				
Occiput posterior	56	5.5	1.48 (1.12–1.96)	1.28 (0.93–1.74)
Occiput anterior	733	3.8	reference	reference
Metabolic acidaemia				
Occiput posterior	2	0.2	1.15 (0.28–4.80)	0.64 (0.08–5.04)
Occiput anterior	33	0.2	reference	reference
Apgar score < 7 at 5 min				
Occiput posterior	20	1.5	2.18 (1.37–3.47)	1.84 (1.11–3.05)
Occiput anterior	188	0.7	reference	reference
Neonatal care				
Occiput posterior	38	2.9	1.87 (1.33–2.62)	1.68 (1.17–2.42)
Occiput anterior	420	1.6	reference	reference
Composite morbidity^b^				
Occiput posterior	47	3.6	1.87 (1.38–2.54)	1.66 (1.19–2.31)
Occiput anterior	520	2.0	reference	reference

Occiput posterior (*n* 1292) and occiput anterior (*n* 26,356). Calculations were only made on deliveries with complete registration of data

Unadsjusted (OR) and adjusted odds ratios (AOR) with 95% confidence intervals (CI)

^a^Adjusted for: for maternal age, nulliparity, pregnancy complication, induction of labour, gestational age at birth, use of epidural analgesia, birth weight and year of delivery

^b^Composite of any of: Apgar score < 7 at 5 min, pH < 7.00, metabolic acidaemia or neonatal care

In Table 4 neonatal outcomes associated with occiput posterior position are stratified by mode of delivery. In spontaneous vaginal occiput posterior delivery the risk of cord pH < 7.10 was increased by 76%, compared with occiput anterior whereas no increased risk was found in operative vaginal or caesarean deliveries. Admission to neonatal care increased two fold with occiput posterior and composite morbidity by 87% in spontaneous vaginal delivery. Neonates with occiput posterior position delivered by caesarean had a decreased risk of admission to neonatal care (AOR 0.54; 95% CI 0.32–0.90) and of composite morbidity (AOR 0.56; 95% CI 0.35–0.90), compared to those with anterior position.

**Table 4:** Neonatal outcomes associated with occiput posterior stratified by mode of delivery

	Spontaneous vaginal		Operative vaginal		Caesarean	
	OR (95% CI)	AOR^a, b^ (95% CI)	OR (95% CI)	AOR^a^ (95% CI)	OR (95% CI)	AOR^a^ (95% CI)
Umbilical artery pH < 7.10	1.57 (1.01–2.42)	1.76 (1.11–2.81)	1.32 (0.81–2.16)	1.11 (0.63–1.98)	0.62 (0.35–1.12)	0.73 (0.38–1.40)
Apgar score < 7 at 5 min	*	*	1.50 (0.67–3.33)	1.20 (0.47–3.06)	0.62 (0.34–1.14)	0.91 (0.48–1.75)
Neonatal care	1.66 (0.88–3.15)	2.01 (1.06–3.81)	1.27 (0.57–2.82)	1.38 (0.59–3.25)	0.45 (0.28–0.71)	0.54 (0.32–0.90)
Composite morbidity^c^	1.50 (0.82–2.76)	1.87 (1.02–3.45)	1.28 (0.68–2.44)	1.26 (0.62–2.56)	0.47 (0.30–0.72)	0.56 (0.35–0.90)

Crude and adjusted odds ratios for posterior versus anterior (reference) position are presented

^a^Adjusted for maternal age, nulliparity, pregnancy complication, induction of labour, gestational age at birth, use of epidural analgesia, birth weight and year of delivery

^b^Spontaneous vaginal delivery also adjusted for duration of pushing

^c^Composite of any of: Apgar score < 7 at 5 min, pH < 7.00, metabolic acidaemia or neonatal care

*No cases among occiput posterior deliveries

Umbilical cord sampling failed or was not performed in 26%. Births with and without sample were compared and there were no differences in admisson to neonatal care or Apgar score less than 4 at 5 min. Among those with a sample, significantly more caesarean sections (7,3% vs. 4,3%, *p* < 0.001) were done and more infants had an Apgar score less than seven at 5 min (0,8% vs. 0,6%, *p* 0.03). Spontaneous onset of labour was significantly more common among those without sample as was the rate of normal pregnancies and of parous women.

The first time period (2004–2007) included 43% of all births in this study. In the first compared to the second period it was significantly less common to induce labour (12,4% vs. 14,9%, *p* < 0.001) and to use epidural (32,1% vs. 33,9%, *p* 0.004) whereas caesarean section was more common (7,3% vs. 6,1% *p* < 0.001). There was no difference in vacuum deliveries.

## Discussion

In a setting where delayed pushing is practiced, there is no increased risk of acidaemia in occiput posterior, compared with occiput anterior deliveries. Occiput posterior delivery is associated with an increased risk of an Apgar score < 7 at 5 min, admission to neonatal care and of composite morbidity. In the analysis stratified by mode of delivery, an increased risk of acidaemia at the level of pH < 7.10, as well as short-term morbidity was found in spontaneous vaginal occiput posterior deliveries, whereas caesarean delivery decreased the need for neonatal care and risk of composite morbidity by 45%, compared with occiput anterior.

This study is limited by the retrospective design and we acknowledge that additional potential confounding factors could have been missed. For example, there was no information about maternal height and body mass index. It has previously been found that obese and short women are at greater risk of having more difficult labours [13–16] and of occiput posterior delivery [4, 5, 17]. Other factors that we were unable to assess and control for were: duration of labour, maternal fever or bleeding during labour, fetal heart rate abnormalities, cord entanglement, and obstetric emergencies. There were also limited data on neonatal outcomes in the database, such as indication for and, duration of neonatal care.

The size of our cohort is a strength to the study. All births took place at the same delivery unit, over a relatively short period of time, which ensured uniformity of labour management. The routine of analysing umbilical artery blood-gases in all deliveries enabled us to investigate the incidence of acidaemia in relation to position at birth. The sampling rate was comparable to what is achieved in other studies. Umbilical cord sampling failed, was not performed or there was no consent in 26%. A sub-analysis on those with and without sample does not indicate that infants with adverse outcomes prevail in the group witout sample. On the contrary, uncomplicated births of parous women were more common.

Consistent with results from a previous and similarly sized cohort study, occiput posterior, compared to occiput anterior, position increased the risk of short-term neonatal morbidity in our study [1]. A few studies have investigated the association between occiput posterior position and cord acidaemia at birth. In a case-control study, no difference in acidaemia (defined as pH < 7.10) was found between occiput anterior and occiput posterior deliveries [18]. Cheng et al. reported that the risk of being born with a pH < 7.00 increased approximately three fold with occiput posterior, compared to occiput anterior position, and in a composite variable that also included base excess less than - 12 mmol/L, the risk increased two fold [1]. In our study, occiput posterior delivery was associated with a pH < 7.10 at birth, had a trend towards an association with pH < 7.05, but had no association at the pH level < 7.00, with or without metabolic acidaemia. However, in the adjusted analysis the association was insignificant even with pH < 7.10. The incidence of persistent occiput posterior position (4.7%), Apgar score < 7 at 5 min (0.8%) and umbilical artery pH < 7.00 (0.4%) was lower in our material compared to the study by Cheng et al. (8.2%; 2.0% and 0.6% respectively). To date, the study by Cheng et al. is, with a cohort of approximately 31,000 deliveries, the largest in this subject area, whereas our cohort consisted of approximately 28,000 deliveries. It is possible that our material was underpowered to detect a difference with regard to those outcomes with low incidences. Though, the estimates were significantly lower in our study.

In the small number of studies that have investigated neonatal outcome in occiput posterior deliveries there is inconsistency in terms of an associated short-term morbidity. Notably high rates of operative vaginal deliveries feature in studies that report on neonatal outcomes, which suggest uncertainty about the applicability of these results to units such as ours, with significantly lower rates [1, 18, 19]. In contrast to previous studies, we found no association between adverse neonatal outcomes and operative vaginal delivery in occiput posterior position [1, 18]. A possible explanatory reason is that forceps are not used in our delivery ward. The greatest contributor in the composite variable was the need for neonatal care, which we were unable to specify. Neonatal care could be related to birth trauma [1, 18], infection [4, 17], or morbidity attributable to meconium [1], which are conditions that have been found to be associated with occiput posterior delivery. There is a need for future studies large enough to confirm or reject the risks of neonatal morbidity identified by previous studies.

There may be several reasons for inconsistency between studies regarding neonatal outcomes and, most likely, differences in labour management are of importance. Practice of delayed or immediate pushing is a possible issue and we speculate that delayed pushing is no disadvantage in occiput posterior position delivery. In studies comparing immediate and delayed pushing, results have been contradictory with regard to benefit and harm to the woman and the neonate. In meta–analyses of randomized controlled trials, where delayed pushing was compared with immediate pushing, there were no difference in neonatal outcomes in terms of Apgar score, admission to intensive care, umbilical artery pH or sepsis [9, 11]. Furthermore, operative vaginal delivery had an average rate of 35% among included studies, meaning that the results may not be generalizable to units where use of operative vaginal delivery is less common and, as yet, studies on the effect of delayed versus immediate pushing from institutions with low intervention rates, as well as on occiput posterior malposition, are lacking.

Most studies report higher caesarean delivery rates in occiput posterior, compared to occiput anterior, deliveries [1–4, 19]. In line with previous studies, our data show a pronounced risk reduction of neonatal morbidity by caesarean delivery of occiput posterior position [1]. Unfortunately, our database held no information on indications for caesarean deliveries, but other studies report dystocia to be the predominating indication with occiput posterior position, in both primipara and multipara [2, 3].

## Conclusion

In a setting with a policy of delayed pushing, delivery in occiput posterior compared with occiput anterior position, increases the risk of short-term neonatal morbidity but is not associated to acidaemia at birth. The significance of the short-term morbidity related to occiput posterior position on subsequent childhood morbidity, and consequences in the long-term, are unknown and need to be investigated in future studies.

## Abbreviations

AOR: Adjusted odds ratio
CI: Confidence interval
OR: Odds ratio
SD: Standard deviation
